# Quantitative Proteomics Identifies the Myb-Binding Protein p160 as a Novel Target of the von Hippel-Lindau Tumor Suppressor

**DOI:** 10.1371/journal.pone.0016975

**Published:** 2011-02-28

**Authors:** Yanlai Lai, Mei Qiao, Meihua Song, Susan T. Weintraub, Yuzuru Shiio

**Affiliations:** 1 Greehey Children's Cancer Research Institute, San Antonio, Texas, United States of America; 2 Department of Biochemistry, The University of Texas Health Science Center, San Antonio, Texas, United States of America; University of Pennsylvania, United States of America

## Abstract

**Background:**

The von Hippel-Lindau (VHL) tumor suppressor gene encodes a component of a ubiquitin ligase complex, which is best understood as a negative regulator of hypoxia inducible factor (HIF). VHL ubiquitinates and degrades the α subunits of HIF, and this is proposed to suppress tumorigenesis and tumor angiogenesis. However, several lines of evidence suggest that there are unidentified substrates or targets for VHL that play important roles in tumor suppression.

**Methodology/Principal Findings:**

Employing quantitative proteomics, we developed an approach to systematically identify the substrates of ubiquitin ligases and using this method, we identified the Myb-binding protein p160 as a novel substrate of VHL.

**Conclusions/Significance:**

A major barrier to understanding the functions of ubiquitin ligases has been the difficulty in pinpointing their ubiquitination substrates. The quantitative proteomics approach we devised for the identification of VHL substrates will be widely applicable to other ubiquitin ligases.

## Introduction

Mutation of the *von Hippel-Lindau* (*VHL)* tumor suppressor gene is associated with a hereditary cancer syndrome called von Hippel-Lindau (VHL) disease, which is characterized by an increased risk of clear cell renal carcinoma, hemangioblastoma of the nervous system, and adrenal pheochromocytoma (for reviews see [1]–[4]). VHL disease patients harbor one wild-type and one defective *VHL* allele while the tumors arising in these patients display somatic inactivation of the remaining wild-type allele. Biallelic *VHL* inactivation is also common in sporadic (non-hereditary) clear cell renal carcinomas and hemangioblastomas. The VHL protein is a component of a protein complex which contains elongin B, elongin C, Cul2, and Rbx1 and this complex functions as an E3 ubiquitin ligase.

VHL is best understood as a negative regulator of hypoxia inducible factor (HIF), a family of transcription factors regulating genes involved in the cellular response to hypoxia. In the presence of oxygen and iron, specific proline residues in HIF become hydroxylated and these hydroxylated prolines are recognized by VHL, which results in ubiquitination and degradation of HIF. Hypoxia or depletion of iron inhibits the prolyl-hydroxylation of HIF, causing stabilization of HIF and induction of HIF target genes such as vascular endothelial growth factor (VEGF) and erythropoietin. Downregulation of HIF by VHL explains some of the phenotypes of tumors with VHL mutations: hemangioblastoma and clear cell renal carcinoma are highly vascular tumors, due at least in part to VEGF overproduction; hemangioblastoma, clear cell renal carcinoma and pheochromocytoma sometimes secrete erythropoietin, leading to overproduction of red blood cells.

However, it is also clear that VHL has functions other than regulating HIF [1]–[4]: 1) VHL was shown to bind to other proteins including fibronectin, atypical PKC family proteins, SP1 transcription factor, RNA polymerase subunits Rpb1 and Rpb7, and a de-ubiquitinating enzyme VDU-1. Among these, VHL ubiquitinates Rpb1 [5], [6] and Rpb7 [7]. 2) There is also evidence that VHL plays HIF-independent roles in extracellular matrix control [8], [9]. 3) Type 2C VHL disease caused by specific VHL mutants such as L188V and V84L predispose mutation carriers to familial pheochromocytomas without hemangioblastomas or renal carcinomas. Importantly, these VHL mutants ubiquitinate and degrade HIF as efficiently as wild-type VHL, which suggests that HIF-independent function(s) of VHL play a role in the generation of pheochromocytomas [9], [10]. 4) Overexpression of constitutively-active HIF in mice did not result in hemangioblastomas or renal carcinomas [11], suggesting that deregulation of HIF is not sufficient to initiate tumors in mice. 5) Gain-of-function HIF-2α mutations were recently identified in familial erythrocytosis patients [12], [13], but these patients did not display predisposition to tumors, suggesting that activation of HIF is not sufficient to induce tumors in humans. These findings suggest that deregulation of HIF is not sufficient for tumorigenesis and that loss of HIF-independent function(s) of VHL plays a critical role in tumorigenesis.

In order to understand the HIF-independent function(s) of VHL, it is important to identify novel VHL substrates/targets. However, identification of substrates of ubiquitin ligases is generally a difficult task because there is no established method to systematically identify the substrates. Employing global protein expression profiling by quantitative proteomics, we devised a strategy to identify the degradation substrates of ubiquitin ligases and using this strategy, identified the Myb-binding protein p160 as a novel substrate of VHL.

## Materials and Methods

### Cell culture

Balb/c3T3, 293T, 786-O, and A498 cells were obtained from ATCC. Balb/c3T3 cells were cultured in Dulbecco's modified Eagle's medium (DMEM) supplemented with 10% fetal calf serum. 293T cells were cultured in DMEM supplemented with 10% calf serum. 786-O cells were cultured in RPMI1640 medium supplemented with 10% fetal calf serum. A498 cells were cultured in Minimum Essential Medium supplemented with 10% fetal calf serum and non-essential amino acids. Calcium phosphate co-precipitation was used for plasmid DNA transfection. MG-132 and desferrioxamine mesylate were purchased from Calbiochem/EMD Biosciences. Mouse VHL siRNA pool (M-040755) and control siRNA pool (D–001206–13) were purchased from Dharmacon and were transfected using Lipofectamine 2000 reagent (Invitrogen). The target sequences for shRNAs are as follows: human VHL, GAGGTCACCTTTGGCTCTTCAGAGA; luciferase, GCACTCTGATTGACAAATACGATTT. VHL and empty vector adenoviruses were generated using AdEasy XL adenoviral vector system (Stratagene).

### Protein sample preparation, ICAT (isotope-coded affinity tag) reagent labeling, and mass spectrometry

Mouse Balb/c3T3 fibroblasts were treated with 100 µM desferrioxamine (DFO, iron chelator) for 24 hours. Soluble protein fraction was prepared as described [14], [15]. As a control, protein sample was also prepared from untreated Balb/c3T3 fibroblasts. 2.5 mg each protein sample was dissolved in the ICAT labeling buffer (0.5% SDS, 6 M urea, 200 mM Tris [pH 8.3], and 5 mM EDTA), reduced with 5 mM Tris(2-carboxyethyl) phosphine (TCEP) for 30 minutes at 37°C, and labeled (desferrioxamine-treated Balb/c3T3 sample: isotopically-heavy ICAT reagent; untreated Balb/c3T3 sample: isotopically-light ICAT reagent). The two labeled samples were combined, proteolyzed to peptides with trypsin, and fractionated by cation-exchange chromatography. ICAT reagent-labeled peptides were purified using the biotin tag present in the reagent and analyzed by microcapillary high performance liquid chromatography-tandem mass spectrometry (μLC-MS/MS) using Thermo Fisher LCQ and LTQ mass spectrometers as described [14], [16]–[18]. Tandem mass spectra were searched against mouse IPI protein database using SEQUEST algorism with a 3Da peptide mass tolerance [19]. Peptide/protein identification was validated by Peptide/ProteinProphet software tools [20], [21]. The ProteinProphet score of 0.5 was used as a cutoff, which corresponds to a false identification rate of 3.5% in iron chelation experiment and 5.3% in p160 co-immunoprecipitation experiment, respectively. Protein abundance ratios were calculated using ASAPRatio software tool [22].

### Immunoprecipitation and immunoblotting

Immunoprecipitation was performed as described [23]. The indicated amounts of cell lysates were separated by SDS-PAGE and were analyzed by immunoblotting as described [18]. Anti-p160 rabbit polyclonal antibody was obtained from Drs. Tom Gonda and Rebecca Keough. Anti-VHL (Ig-32) was purchased from BD Pharmingen. Anti-VHL (FL-181) was purchased from Santa Cruz Biotechnology. Anti-FLAG M2 and anti-tubulin mouse monoclonal antibodies were purchased from Sigma. Anti-hydroxyproline antibody was purchased from Advanced Targeting Systems. Far-western blotting was performed as described [24].

### In vitro ubiquitination assay

VHL-null 786-O cells were washed twice with cold hypotonic extraction buffer (20 mM Tris (pH 7.5), 5 mM KCl, 1.5 mM MgCl_2_, 1 mM dithiothreitol) and the cells were disrupted in a Dounce homogenizer. Following lysis, crude extract was centrifuged at 10,000×*g* for 10 minutes at 4°C and stored in aliquots at -80°C. Biotinylated p160 was in vitro translated using TNT coupled transcription/translation system and Transcend™ tRNA (Promega). Ubiquitination assays were performed at 30°C in a total volume of 40 µl, containing 2 µl of in vitro translated, biotinylated p160, 27 µl of 786-O cell extract, 4 µl of 10×ATP-regenerating system (20 mM Tris, pH 7.5, 10 mM ATP, 10 mM magnesium acetate, 300 mM creatine phosphate, 0.5 mg/ml creatine phosphokinase), 4 µl of 5 mg/ml ubiquitin (where indicated), and 0.83 µl of 150 µM ubiquitin aldehyde (Biomol International). Where indicated, 200 ng of purified GST-VHL was preincubated with the reaction mixture at room temperature for 5 min prior to the addition of the substrate. Aliquots were removed at indicated times, mixed with SDS-PAGE sample buffer, and analyzed by SDS-PAGE and protein blotting. The detection of the blot was carried out using HRP-conjugated Streptavidin (Invitrogen).

## Results

### Quantitative proteomic analysis of iron chelation-induced protein expression changes

**Figure 1:**
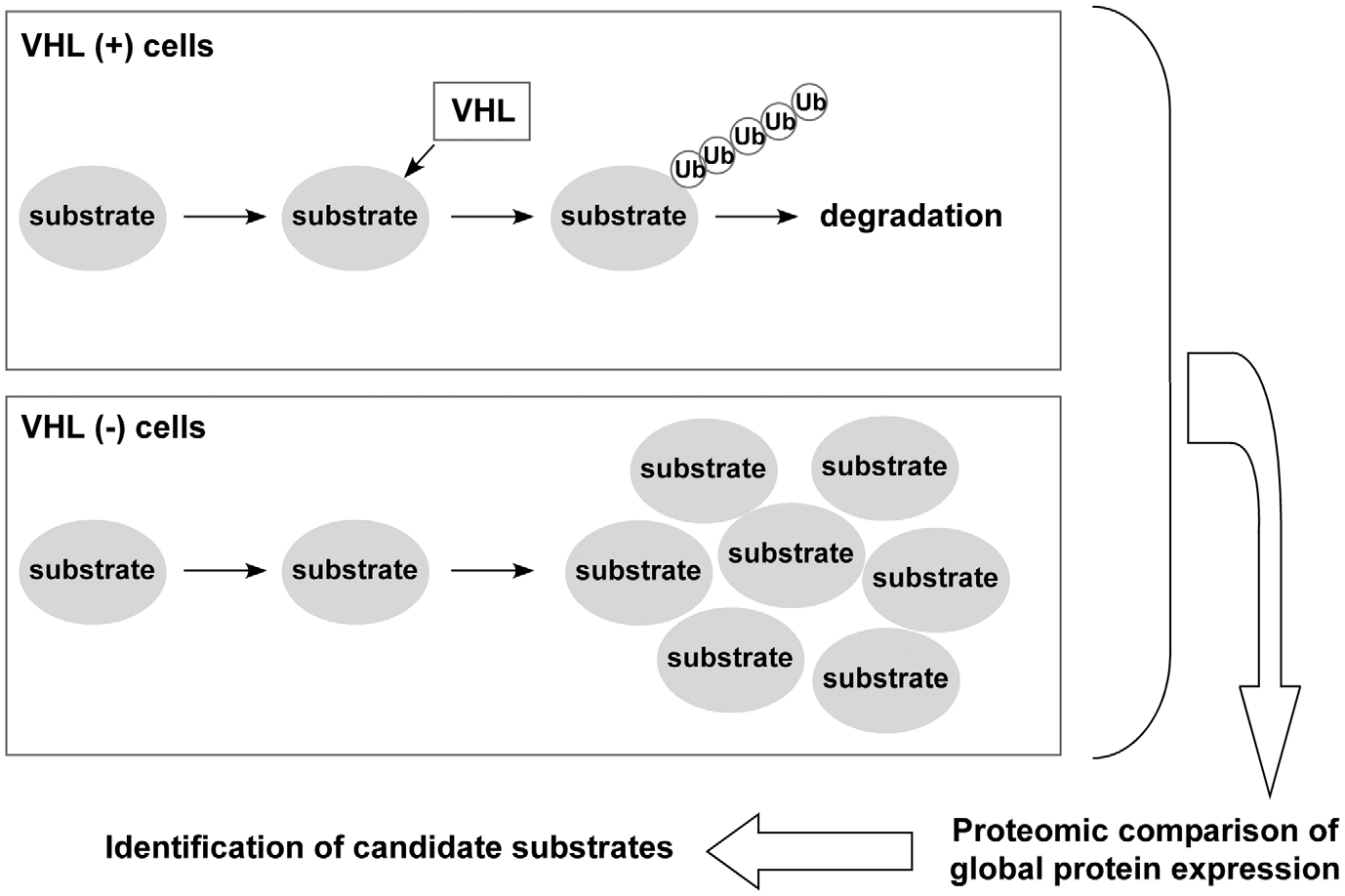
Outline of proteomic screen for VHL substrates. In cells with functional VHL, VHL(+), VHL substrates are ubiquitinated and degraded by the proteasome. In cells without functional VHL, VHL(-), VHL substrates accumulate. By comparing the global protein expression of VHL(+) and VHL(-) cells by quantitative proteomics, candidate VHL substrates can be identified.

To identify novel ubiquitination substrates of VHL, we undertook a proteomic screening using ICAT (isotope-coded affinity tag) quantitative proteomics technology [16], [25]. Unlike other isotope-labeling proteomics approaches such as SILAC and iTRAQ, the ICAT procedure selects only cysteine-containing peptides (Note that 96% of all human proteins contain at least one cysteine) and thus effectively reduces the complexity of peptide mixtures, allowing sensitive detection of low-abundance proteins. Since the VHL ubiquitin ligase catalyzes the formation of lysine-48-linked poly-ubiquitin chains which target proteins for proteasomal degradation, we reasoned that the VHL substrates would accumulate in cells that do not have functional VHL, which can be detected by comparing the global protein expression in cells with and without functional VHL (Figure 1). Because the ubiquitination and degradation of HIF by VHL can be inhibited by iron chelation, we used iron chelation to inhibit protein ubiquitination by VHL and analyzed the resulting protein expression changes.

Mouse Balb/c3T3 fibroblasts were treated with an iron chelator, desferrioxamine, at 100 µM for 24 hours or left untreated and the cell lysates were prepared. 2.5 mg each cell lysate was analyzed for protein expression by the ICAT approach. The resulting dataset was subjected to statistical analysis [20], [21] and at a ProteinProphet probability score of 0.5 or higher (corresponding to a false identification rate of 3.5%), 612 proteins were identified and quantified. A partial list of protein changes induced by iron chelation is shown in Table 1 (For complete lists of proteins displaying more than 2-fold induction or reduction upon iron chelation, see Tables S1 and S2). As expected, we determined that HIF-3α as well as a number of HIF transcriptional targets is induced by iron chelation. In addition, we also found other protein changes such as upregulation of Myb-binding protein p160 and downregulation of different mitochondrial proteins.

p160 was originally identified as a predominantly nucleolar protein that binds to the negative regulatory domain of c-Myb [26]. More recently p160 was shown to bind and inhibit the coactivator PGC-1α [27], which results in downregulation of gene expression of mitochondrial proteins and mitochondrial respiration. Therefore, the reduced expression of several mitochondrial proteins upon iron chelation (Table 1) may be due to upregulation of p160. In the following sections, we investigated the possibility that p160 is a degradation substrate of VHL.

**Table 1:** Partial list of protein expression changes upon iron chelation.

Protein name	(+)DFO : (-)DFO^a^
HIF-3α	2.75∶1
Myb-binding protein p160	2.70∶1
Upregulation of HIF targets	
ER oxidoreductase 1-like	2.94∶1
Lysyl oxidase	2.78∶1
NDRG1	2.50∶1
Lactate dehydrogenase A	2.50∶1
Calgizzarin	2.27∶1
CYR61/CCN1	2.08∶1
Phosphoglycerate kinase 1	2.04∶1
Triosephosphate isomerase	2.04∶1
Downregulation of mitochondrial proteins	
Mitochondrial processing peptidase α	0.15∶1
Cytochrome b-c1 complex subunit 1	0.25∶1
2-amino-3-ketobutyrate coenzyme A ligase	0.42∶1
Electron transfer flavoprotein	0.43∶1
Cytochrome b-c1 complex subunit 6	0.46∶1

a Relative abundance of each protein in Balb/c3T3 cells with and without desferrioxamine treatment [(+) and (-) DFO] is shown.

### Degradation of p160 by VHL

**Figure 2:**
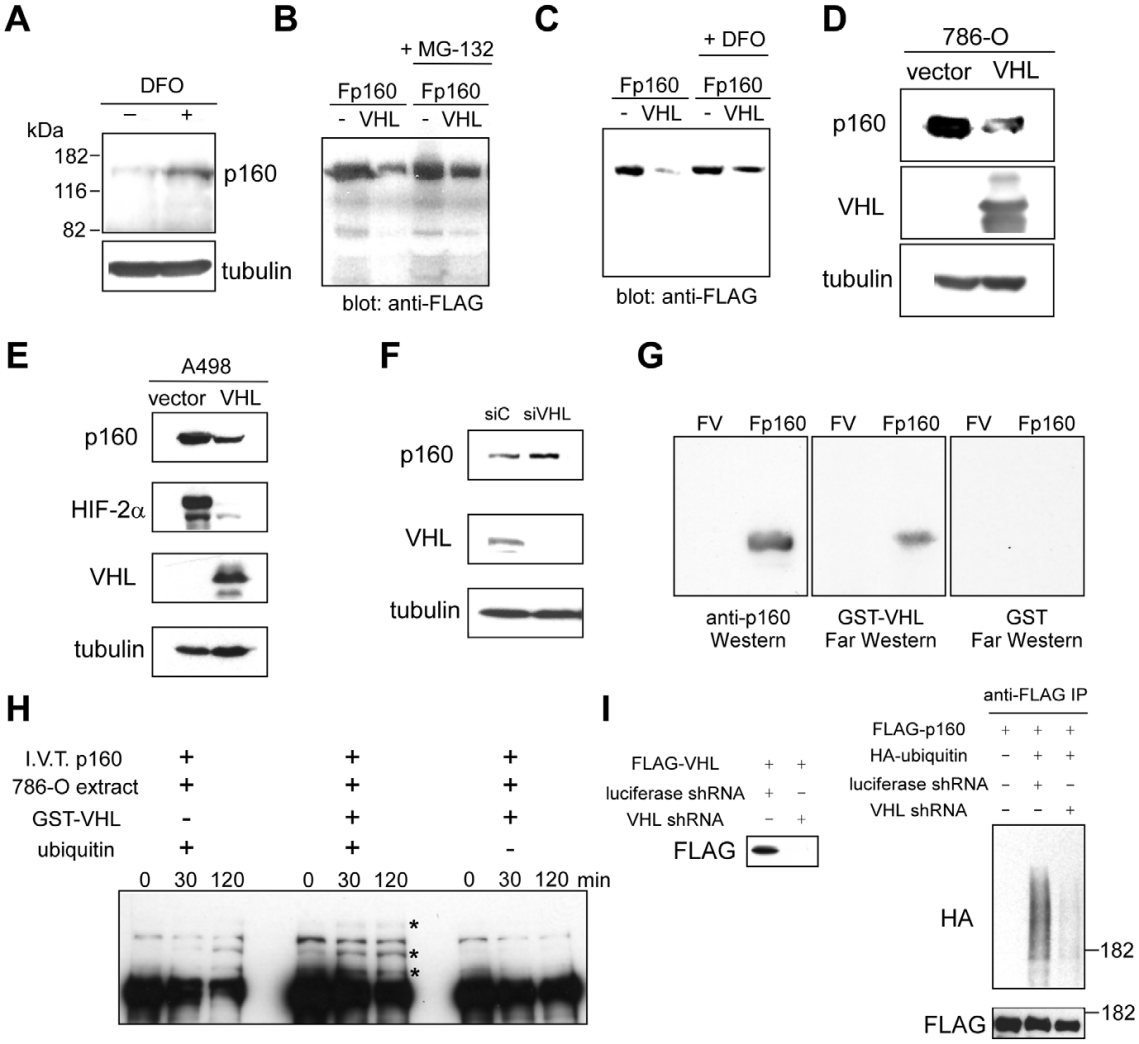
Degradation of p160 by VHL. (A) Increased p160 protein expression upon iron chelation. Mouse Balb/c3T3 fibroblasts were treated with or without 100 µM desferrioxamine (DFO, iron chelator) for 24 hours and the p160 and tubulin expression was analyzed by immunoblotting using 30 µg whole cell lysate. (B) VHL degrades p160 by a proteasome-dependent mechanism. 293T cells were transfected with FLAG-p160 with or without VHL. Where indicated, the transfected cells were treated with 10 µM MG-132 for 12 hours. The expression of FLAG-p160 was examined by immunoblotting using 30 µg whole cell lysate. (C) Degradation of p160 by VHL is abolished by iron chelation. 293T cells were transfected with FLAG-p160 with or without VHL. Where indicated, the transfected cells were treated with 100 µM desferrioxamine (DFO) for 12 hours. The expression of FLAG-p160 was examined by immunoblotting using 30 µg whole cell lysate. (D) Degradation of endogenous p160 by VHL in 786-O cells. VHL-null 786-O renal carcinoma cells were infected with VHL or empty adenovirus vector and 48 hours after infection, the expression of p160, VHL, and tubulin was analyzed by immunoblotting using 30 µg whole cell lysate. (E) Degradation of endogenous p160 by VHL in A498 cells. VHL-null A498 renal carcinoma cells were infected with VHL or empty adenovirus vector and 48 hours after infection, the expression of p160, HIF-2α, VHL, and tubulin was analyzed by immunoblotting using 30 µg whole cell lysate. (F) Effect of VHL knockdown on p160 expression. Balb/c3T3 cells were transfected with control or VHL siRNA and 72 hours after transfection, the expression of p160, VHL, and tubulin was analyzed by immunoblotting using 30 µg whole cell lysate. (G) VHL directly binds p160. FLAG-p160 was immunoprecipitated from transfected 293T cells (FLAG empty vector as control), fractionated by SDS-PAGE, and transferred to nitrocellulose membrane. Triplicate protein blots were probed with anti-p160 antibody (left), bacterially-produced purified GST-VHL (middle), or GST (right). (H) In vitro ubiquitination of p160 by VHL. In vitro translated p160 was incubated at 30°C with the extract of VHL-null 786-O cells and ATP-regenerating system in the presence or absence of GST-VHL and ubiquitin as indicated. Aliquots were removed at indicated times and were analyzed for protein ubiquitination. 0 minute samples were removed after assembling the reaction mixture at room temperature without incubation at 30°C. Ubiquitinated p160 is indicated by asterisks. (I) Effect of VHL knockdown on p160 ubiquitination in vivo. Left: 293T cells were transfected with FLAG-VHL and luciferase shRNA or VHL shRNA expression vector and 48 hours after transfection, the expression of FLAG-VHL was analyzed by anti-FLAG immunoblotting. Right: 293T cells were transfected with FLAG-p160, HA-ubiquitin, luciferase shRNA, or VHL shRNA where indicated, and FLAG-p160 was immunoprecipitated by anti-FLAG antibody and was analyzed by anti-HA or anti-FLAG immunoblotting.

We confirmed the upregulation of p160 upon iron chelation by immunoblotting (Figure 2A). Furthermore, co-expression of VHL dramatically reduced the expression levels of FLAG-tagged-p160 in 293T cells and this was abolished by a proteasome inhibitor, MG-132, suggesting that VHL induces proteasome-dependent degradation of p160 (Figure 2B). Degradation of FLAG-p160 by VHL was also abolished by iron chelation with desferrioxamine (DFO) (Figure 2C), indicating that degradation of p160 by VHL requires iron as previously demonstrated for HIF. When VHL expression was restored in VHL-null 786-O renal carcinoma cells or A498 renal carcinoma cells, VHL degraded endogenous p160 (Figure 2D and E). Conversely, siRNA-mediated knockdown of VHL expression in Balb/c3T3 cells resulted in stabilization of endogenous p160 (Figure 2F). Furthermore, specific and direct binding of VHL and p160 was demonstrated by probing the blot of immunoprecipitated FLAG-p160 with bacterially-produced purified GST-VHL using Far-western technique (Figure 2G).

**Figure 3:**
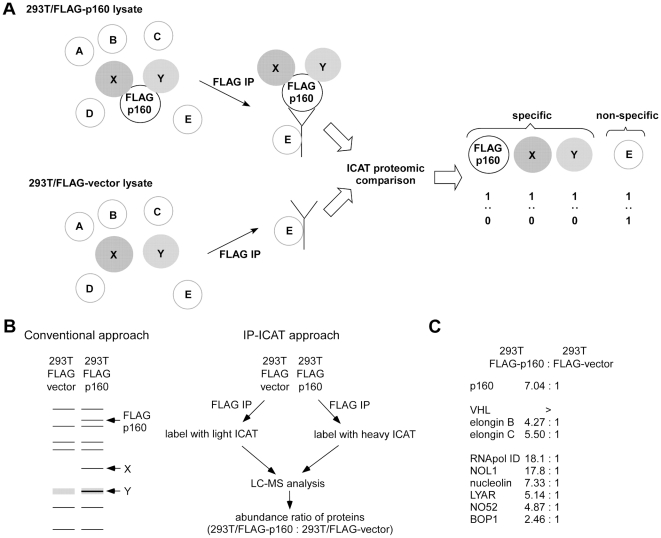
Proteomic analysis of p160-interacting proteins. (A) Outline of the IP-ICAT approach. To identify p160-interacting proteins, FLAG-p160 is immunoprecipitated from transfected 293T cells, which results in isolation of FLAG-p160, its associated proteins “X” and “Y,” and non-specifically contaminating protein “E.” As a control, lysate of 293T cells transfected with empty FLAG vector is also immunoprecipitated, which isolates non-specifically contaminating protein “E.” The relative abundance of the protein components in the two immunoprecipitates can be determined by the ICAT quantitative proteomics approach. The specific components of the FLAG-p160 complex display enrichment in the FLAG-p160 IP sample whereas non-specific contaminant does not. In this way, specific components of the protein complex can be distinguished from non-specific contaminant. (B) Comparison of the conventional SDS-PAGE-based approach and IP-ICAT approach for the analysis of protein complexes. In the conventional approach, immunopurified protein complex is fractionated by SDS-PAGE and the protein bands specific to test IP sample (absent in control IP sample) are excised and analyzed for peptide sequence determination. The protein ‘Y’ is specific to test IP sample, but can be missed by this approach due to a co-migrating background band. In the IP-ICAT approach, the components of the test IP and control IP samples are compared without using a gel. (C) Partial list of proteins identified in the p160 complex. Components of the VHL E3 ligase complex as well as several nucleolar proteins were identified as p160-interacting proteins. VHL displayed obvious enrichment in the FLAG-p160 IP sample, but was difficult to quantify, which is indicated by “>”.

The proteomic analysis of the p160-containing protein complex also confirmed the physical interaction of p160 and the VHL ubiquitin ligase complex (Figure 3): FLAG-p160 was transfected into 293T cells and the protein complex containing FLAG-p160 was purified by anti-FLAG immunoprecipitation under non-denaturing conditions. As a control, we used 293T cells transfected with FLAG empty vector. The protein components in the two immunoprecipitation samples (FLAG-p160 and FLAG-vector) were compared by the ICAT approach and the specific components of the FLAG-p160 complex were identified by their increased abundance in the FLAG-p160 immunoprecipitate compared with FLAG-vector immunoprecipitate (Figure 3A). This analysis identified the co-immunoprecipitation of FLAG-p160 with the components of the VHL ubiquitin ligase complex (VHL, elongin B, and elongin C) as well as a number of nucleolar proteins (Figure 3C, for a complete list of proteins displaying more than 2-fold enrichment in FLAG-p160 immunoprecipitate, see Table S3). Collectively, these results suggest that VHL induces proteasome- and iron-dependent degradation of p160 through direct physical interaction.

**Figure 4:**
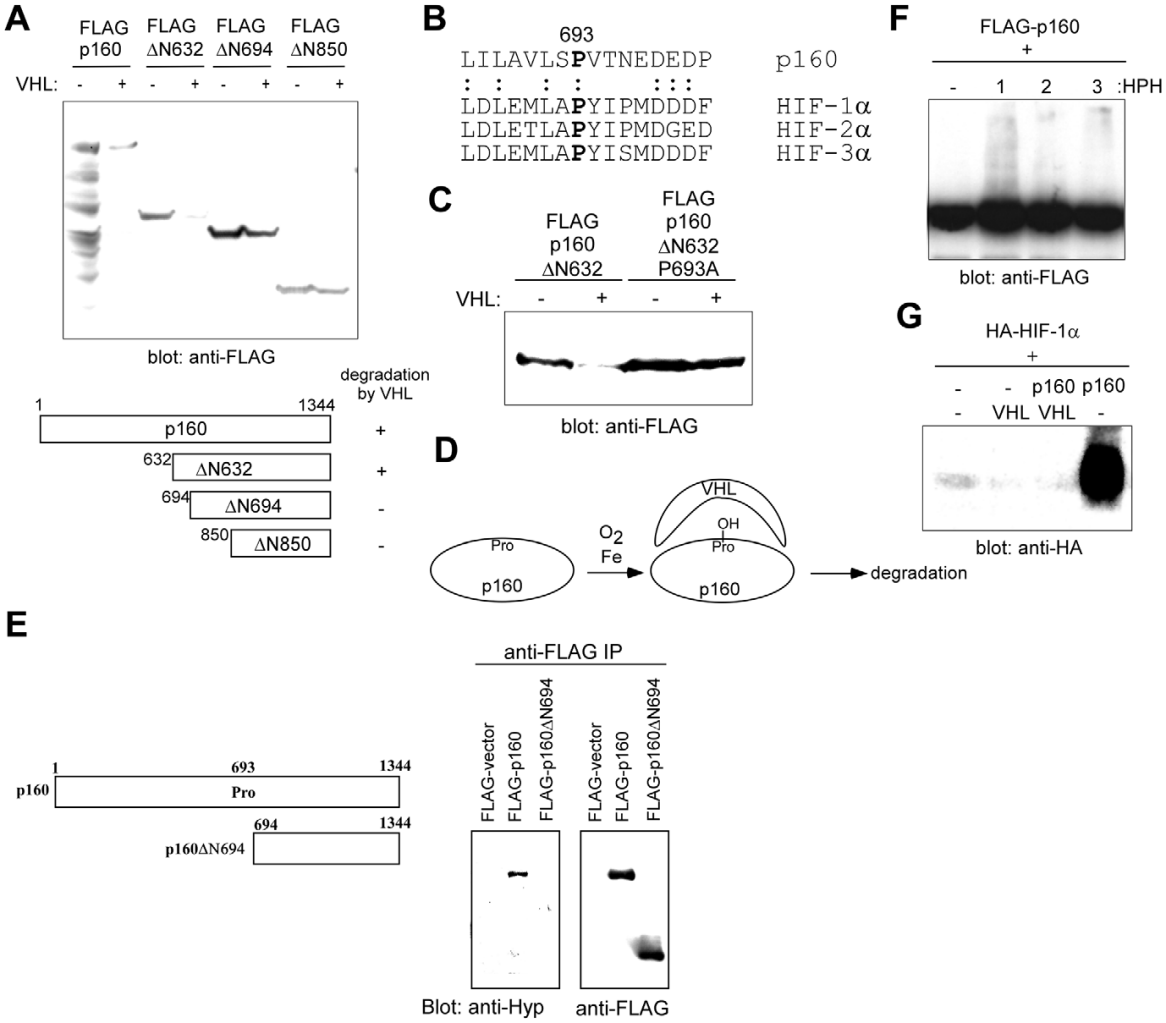
Mechanism of p160 degradation by VHL. (A) Deletion mapping of the p160 degron. A series of N-terminal p160 deletion mutants (diagram shown below) was analyzed for their sensitivity to VHL-mediated degradation in transfected 293T cells by immunoblotting. (B) p160 has a motif similar to the one surrounding the hydroxylated proline in HIFs. Alignment of p160 amino acid 686-701 and the C-terminal oxygen-dependent degradation domain (ODD) of HIF-1α, HIF-2α, and HIF-3α is shown. (C) Proline 693 is critical for degradation of p160 by VHL. The effect of co-transfection of VHL on FLAG-p160ΔN632 or FLAG-p160ΔN632P693A was analyzed in 293T cells. Whereas p160 ΔN632 was efficiently degraded by VHL, mutation of proline 693 to alanine abolished degradation by VHL. (D) Model for the degradation of p160 by VHL. (E) Prolyl hydroxylation of p160. FLAG-p160 or FLAG-p160ΔN694 was transfected into 293T cells and was analyzed by anti-FLAG immunoprecipitation followed by anti-hydroxyproline or anti-FLAG immunoblotting. (F) Effect of prolyl hydroxylases on p160. 293T cells were transfected with FLAG-p160 alone or in conjunction with HIF prolyl hydroxylase HPH1, 2, or 3, and p160 expression was analyzed by anti-FLAG immunoblotting using 30 µg whole cell lysate. (G) Stabilization of HIF-1α by p160. 293T cells were transfected with HA-HIF-1α alone or together with p160 and/or VHL as indicated and the HA-HIF-1α expression was analyzed by anti-HA immunoblotting using 30 µg whole cell lysate.

To test whether VHL can ubiquitinate p160, we conducted in vitro ubiquitination assays following a published procedure [28], [29]. In vitro translated p160 was incubated with cell extract of VHL-null 786-O cells. Where indicated, 786-O cell extract was supplemented with bacterially-produced purified GST-VHL and/or ubiquitin. As shown in Figure 2H, while the 786-O cell extract, which lacks functional VHL, induced modest ubiquitination of p160, addition of GST-VHL to the 786-O extract resulted in more robust ubiquitination of p160 (ubiquitinated p160 is indicated by asterisks).This suggests that VHL can induce ubiquitination of p160 in vitro. Ubiquitination of p160 by VHL-null 786-O cell extract also suggests the existence of other ubiquitin ligase(s) that can ubiquitinate p160, which might explain the relatively modest stabilization of p160 upon VHL knockdown (Figure 2F). We then analyzed the role of VHL in p160 ubiquitination in vivo. 293T cells were co-transfected with FLAG-p160 and HA-ubiquitin and ubiquitination of p160 was assessed by anti-FLAG immunoprecipitation followed by anti-HA immunoblotting. As shown in Figure 2I right, VHL shRNA, which can efficiently knock down VHL expression (Figure 2I left), abolished ubiquitination of FLAG-p160, suggesting that VHL mediates ubiquitination of p160 in vivo. Although it remains possible that VHL indirectly mediates p160 ubiquitination, these results, together with the direct binding of VHL and p160 (Figure 2G) as well as the prolyl hydroxylation of p160 (Figure 4E), suggest that p160 is a ubiquitination substrate of VHL.

### Mechanism of p160 degradation by VHL

We then mapped the p160 domain(s) necessary for degradation by VHL using a series of N-terminal deletion mutants of p160 (Figure 4A). An N-terminal deletion mutant to amino acid 632 (ΔN632) was still degraded by VHL, but an N-terminal deletion mutant to amino acid 694 (ΔN694) was no longer degraded by VHL. This suggests that there is a degron between amino acid 632 and 694 of p160 (Figure 4A). We noticed that this region of p160 contains a sequence motif similar to the one surrounding the hydroxylated proline in HIF-α (Figure 4B). Importantly, mutation of this proline residue (Pro693) to alanine (ΔN632 P693A) abolished degradation by VHL (Figure 4C), consistent with the notion that (hydroxylated) Pro693 is recognized by VHL for ubiquitination. P693A substitution in the context of full length p160 did not completely abolish degradation by VHL (data not shown), suggesting that there are additional degron(s) in p160 that are targeted by VHL.

Our current working hypothesis is that Pro693 of p160 is hydroxylated in the presence of iron and oxygen, which results in ubiquitination by VHL and degradation by the proteasome (Figure 4D). To determine whether p160 can be prolyl hydroxylated in vivo, 293T cells were transfected with FLAG-p160 or FLAG-p160 ΔN694, which lacks N-terminal 693 amino acids of p160 including Pro693. FLAG-p160 or FLAG-p160 ΔN694 was immunoprecipitated by anti-FLAG antibody and was analyzed by anti-hydroxyproline or anti-FLAG immunoblotting. As shown in Figure 4E, FLAG-p160, but not FLAG-p160NΔ694, was detected by anti-hydroxyproline antibody, demonstrating the prolyl hydroxylation of FLAG-p160. Consistent with the role of prolyl hydroxylation in p160 ubiquitination, we found that HIF prolyl hydroxylases (HPH1 and to a lesser extent HPH2 and 3) induce laddering and smearing of the p160 protein band, which is indicative of ubiquitination (Figure 4F). In addition, we also found that co-expression of p160 dramatically stabilizes HIF-1α in 293T cells (Figure 4G, lane 4), which is normally very unstable due to VHL-mediated degradation. Stabilization of HIF-1α by p160 was abolished by coexpression of VHL (Figure 4G, lane 3). Stabilization of HIF-1α by p160 may be due to the titration of VHL or HPHs.

## Discussion

Our ICAT proteomic analysis identified the Myb-binding protein p160 as a protein whose expression is induced upon iron chelation, and further analyses demonstrated that p160 is a ubiquitination substrate of VHL. VHL directly binds and degrades p160 in an iron-dependent and proteasome-dependent manner.

p160 is a transcriptional co-repressor of the PGC-1α/NRF-1 transcription complex which controls the coordinated expression of genes essential for mitochondrial function [27]. Interestingly, Hervouet *et al.* reported that restoration of wild-type VHL expression in VHL-deficient 786-O renal carcinoma cells results in increased oxidative phosphorylation (OXPHOS) protein expression and enhanced mitochondrial respiratory chain activities [30], which may be mediated by degradation of p160 by VHL. In tumors with VHL mutation, p160 would be stabilized and inhibit the expression of mitochondrial OXPHOS proteins, leading to reduced mitochondrial respiration and concomitantly increased glycolytic ATP production. This could be a molecular basis for the well known “Warburg effect” (reprogramming of tumor metabolism from oxidative to glycolytic metabolism, for a review see [31]) in VHL-mutated tumors.

Among the mitochondrial protein genes regulated by the PGC-1α/NRF-1 transcription complex is cytochrome c [32], which is a central mediator of apoptosis. Hence, mutation of VHL may result in upregulation of p160 and protection against apoptosis, which would provide a selective advantage to cancer cells. In fact, several studies demonstrated that VHL sensitizes cells to apoptosis induction by different stimuli such as TNF-α, DNA damage, and NGF withdrawal [33]–[35]. Furthermore, forced overexpression of VHL in VHL-null 786-O cells was shown to induce apoptosis [36]. Restoration of VHL expression in VHL-null 786-O renal cells also results in reduced tumor growth in nude mice [36], [37]. VHL may suppress tumors by sensitizing cells to apoptosis through downregulation of p160.

We employed a quantitative proteomics approach to analyze the components of the p160 complex (Figure 3). Conventional approach for analyzing the components of protein complex is to fractionate the immunopurified protein complex along with a control purification sample by SDS-PAGE, excise the protein bands specific to the protein complex sample, and determine their identities by mass spectrometry (Figure 3B, left). Although this approach is widely used and allows sensitive detection of protein complex components, each band has to be analyzed separately and some protein complex components can be missed due to gel background: In the example shown in Figure 3B, left, protein Y is specific to FLAG-p160 complex, but it may not be identified because of a co-migrating background band. On the contrary, the use of ICAT quantitative proteomics to compare specific and control IP samples (IP-ICAT approach, Figure 3B, right) allows simultaneous detection of multiple protein complex components without using a gel. Gentle purification conditions would more likely preserve unstable protein-protein interactions, but may also yield higher purification background, which creates a problem for gel-based analysis. The gel-free IP-ICAT approach is not affected by purification background and allows the use of more gentle purification conditions. Using this IP-ICAT approach, we were able to show that p160 interacts with endogenous VHL ubiquitin ligase complex (Figure 3C).

The quantitative proteomics approach we devised for the identification of VHL substrates (Figure 1) will be widely applicable to other ubiquitin ligases. It is estimated that there are several hundred E3 ubiquitin ligases encoded in the human genome, but in most cases, their ubiquitination substrates are poorly characterized. Ubiquitin ligases and their substrates often do not interact stably, which has been hampering the identification of ligase-substrate pairs. Our protein expression profiling approach allows the identification of candidate substrate proteins by their increased abundance in cells without functional ubiquitin ligase compared with cells that retain the ubiquitin ligase. These candidates can then be verified by biochemical and molecular biological analyses. Although mass spectrometry-based quantitative proteomics cannot currently detect every protein in mammalian cells [25], proteomic analysis of sub-proteomes such as subcellular fractions can increase the proteome coverage by quantitative proteomics. Protein expression profiling in the subcellular compartment where a given ubiquitin ligase resides would enable more sensitive detection of its ubiquitination substrates. Further refinement of proteomics-based screen we presented here will allow the identification of ubiquitination substrates for many other ubiquitin ligases.

## Supporting Information

Table S1 **List of proteins displaying more than 2-fold induction upon iron chelation.**
(XLS)

Table S2 **List of proteins displaying more than 2-fold reduction upon iron chelation.** The list of proteins induced or reduced by more than 2-fold upon iron chelation is available as ‘list of proteins induced by iron chelation.xls’ and ‘list of proteins reduced by iron chelation.xls’ files, respectively. These datasets contain proteins with ProteinProphet probability score≥0.5. Protein abundance ratios were calculated using ASAPRatio software tool. The description of each column in these data files is as follows: A: ProteinProphet probability score B: Protein abundance ratio (desferrioxamine (+)/desferrioxamine (-)) C: Protein name
(XLS)

Table S3 **List of proteins coimmunoprecipitated with p160. **The list of proteins that displayed more than 2-fold enrichment in FLAG-p160 immunoprecipitate is available as ‘list of proteins coimmunoprecipitated with p160.xls’ file. This dataset contains proteins with ProteinProphet probability score≥0.5. Protein abundance ratios were calculated using ASAPRatio software tool. Abundance ratio of 999 denotes that the protein displayed obvious enrichment in the FLAG-p160 immunoprecipitate, but was difficult to quantify. The description of each column in this data file is as follows: A: ProteinProphet probability score B: Protein abundance ratio (FLAG-p160/FLAG-vector) C: Protein name
(XLS)

## References

[1] Kaelin WG (2005). The von Hippel-Lindau protein, HIF hydroxylation, and oxygen sensing.. Biochem Biophys Res Commun.

[2] Kim WY, Kaelin WG (2004). Role of VHL gene mutation in human cancer.. J Clin Oncol.

[3] Barry RE, Krek W (2004). The von Hippel-Lindau tumour suppressor: a multi-faceted inhibitor of tumourigenesis.. Trends Mol Med.

[4] Czyzyk-Krzeska MF, Meller J (2004). von Hippel-Lindau tumor suppressor: not only HIF's executioner.. Trends Mol Med.

[5] Kuznetsova AV, Meller J, Schnell PO, Nash JA, Ignacak ML (2003). von Hippel-Lindau protein binds hyperphosphorylated large subunit of RNA polymerase II through a proline hydroxylation motif and targets it for ubiquitination.. Proc Natl Acad Sci U S A.

[6] Mikhaylova O, Ignacak ML, Barankiewicz TJ, Harbaugh SV, Yi Y (2008). The von Hippel-Lindau tumor suppressor protein and Egl-9-Type proline hydroxylases regulate the large subunit of RNA polymerase II in response to oxidative stress.. Mol Cell Biol.

[7] Na X, Duan HO, Messing EM, Schoen SR, Ryan CK (2003). Identification of the RNA polymerase II subunit hsRPB7 as a novel target of the von Hippel-Lindau protein.. Embo J.

[8] Bishop T, Lau KW, Epstein AC, Kim SK, Jiang M (2004). Genetic analysis of pathways regulated by the von hippel-lindau tumor suppressor in Caenorhabditis elegans.. PLoS Biol.

[9] Hoffman MA, Ohh M, Yang H, Klco JM, Ivan M (2001). von Hippel-Lindau protein mutants linked to type 2C VHL disease preserve the ability to downregulate HIF.. Hum Mol Genet.

[10] Clifford SC, Cockman ME, Smallwood AC, Mole DR, Woodward ER (2001). Contrasting effects on HIF-1alpha regulation by disease-causing pVHL mutations correlate with patterns of tumourigenesis in von Hippel-Lindau disease.. Hum Mol Genet.

[11] Kim WY, Safran M, Buckley MR, Ebert BL, Glickman J (2006). Failure to prolyl hydroxylate hypoxia-inducible factor alpha phenocopies VHL inactivation in vivo.. Embo J.

[12] Percy MJ, Furlow PW, Lucas GS, Li X, Lappin TR (2008). A gain-of-function mutation in the HIF2A gene in familial erythrocytosis.. N Engl J Med.

[13] Percy MJ, Beer PA, Campbell G, Dekker AW, Green AR (2008). Novel exon 12 mutations in the HIF2A gene associated with erythrocytosis.. Blood.

[14] Shiio Y, Eisenman RN, Yi EC, Donohoe S, Goodlett DR (2003). Quantitative proteomic analysis of chromatin-associated factors.. J Am Soc Mass Spectrom.

[15] Juin P, Hueber AO, Littlewood T, Evan G (1999). c-Myc-induced sensitization to apoptosis is mediated through cytochrome c release.. Genes Dev.

[16] Gygi SP, Rist B, Gerber SA, Turecek F, Gelb MH (1999). Quantitative analysis of complex protein mixtures using isotope-coded affinity tags.. Nat Biotechnol.

[17] Han DK, Eng J, Zhou H, Aebersold R (2001). Quantitative profiling of differentiation-induced microsomal proteins using isotope-coded affinity tags and mass spectrometry.. Nat Biotechnol.

[18] Shiio Y, Donohoe S, Yi EC, Goodlett DR, Aebersold R (2002). Quantitative proteomic analysis of Myc oncoprotein function.. Embo J.

[19] Eng J, McCormack AL, Yates JR (1994). An approach to correlate tandem mass spectral data of peptides with amino acid sequences in a protein database.. Journal for the American Society for Mass Spectrometry.

[20] Keller A, Nesvizhskii AI, Kolker E, Aebersold R (2002). Empirical statistical model to estimate the accuracy of peptide identifications made by MS/MS and database search.. Anal Chem.

[21] Nesvizhskii AI, Keller A, Kolker E, Aebersold R (2003). A statistical model for identifying proteins by tandem mass spectrometry.. Anal Chem.

[22] Li XJ, Zhang H, Ranish JA, Aebersold R (2003). Automated statistical analysis of protein abundance ratios from data generated by stable-isotope dilution and tandem mass spectrometry.. Anal Chem.

[23] Shiio Y, Eisenman RN (2003). Histone sumoylation is associated with transcriptional repression.. Proc Natl Acad Sci U S A.

[24] Ohh M, Park CW, Ivan M, Hoffman MA, Kim TY (2000). Ubiquitination of hypoxia-inducible factor requires direct binding to the beta-domain of the von Hippel-Lindau protein.. Nat Cell Biol.

[25] Shiio Y, Aebersold R (2006). Quantitative proteome analysis using isotope-coded affinity tags and mass spectrometry.. Nature Protocols.

[26] Tavner FJ, Simpson R, Tashiro S, Favier D, Jenkins NA (1998). Molecular cloning reveals that the p160 Myb-binding protein is a novel, predominantly nucleolar protein which may play a role in transactivation by Myb.. Mol Cell Biol.

[27] Fan M, Rhee J, St-Pierre J, Handschin C, Puigserver P (2004). Suppression of mitochondrial respiration through recruitment of p160 myb binding protein to PGC-1alpha: modulation by p38 MAPK.. Genes Dev.

[28] Cockman ME, Masson N, Mole DR, Jaakkola P, Chang GW (2000). Hypoxia inducible factor-alpha binding and ubiquitylation by the von Hippel-Lindau tumor suppressor protein.. J Biol Chem.

[29] Choi SM, Choi KO, Park YK, Cho H, Yang EG (2006). Clioquinol,a Cu(II)/Zn(II) chelator inhibits both ubiquitination and asparagine hydroxylation of HIF-1alpha, leading to expression of VEGF and EPO in normoxic cells..

[30] Hervouet E, Demont J, Pecina P, Vojtiskova A, Houstek J (2005). A new role for the von Hippel-Lindau tumor suppressor protein: stimulation of mitochondrial oxidative phosphorylation complex biogenesis.. Carcinogenesis.

[31] Dang CV, Semenza GL (1999). Oncogenic alterations of metabolism.. Trends Biochem Sci.

[32] Morrish F, Giedt C, Hockenbery D (2003). c-MYC apoptotic function is mediated by NRF-1 target genes.. Genes Dev.

[33] Qi H, Ohh M (2003). The von Hippel-Lindau tumor suppressor protein sensitizes renal cell carcinoma cells to tumor necrosis factor-induced cytotoxicity by suppressing the nuclear factor-kappaB-dependent antiapoptotic pathway.. Cancer Res.

[34] Lee S, Nakamura E, Yang H, Wei W, Linggi MS (2005). Neuronal apoptosis linked to EglN3 prolyl hydroxylase and familial pheochromocytoma genes: developmental culling and cancer.. Cancer Cell.

[35] Roe JS, Kim H, Lee SM, Kim ST, Cho EJ (2006). p53 stabilization and transactivation by a von Hippel-Lindau protein.. Mol Cell.

[36] Kim M, Yan Y, Lee K, Sgagias M, Cowan KH (2004). Ectopic expression of von Hippel-Lindau tumor suppressor induces apoptosis in 786-O renal cell carcinoma cells and regresses tumor growth of 786-O cells in nude mouse.. Biochem Biophys Res Commun.

[37] Iliopoulos O, Kibel A, Gray S, Kaelin WG (1995). Tumour suppression by the human von Hippel-Lindau gene product.. Nat Med.

